# Supplementary material for: Content-rich biological network constructed by mining PubMed abstracts
Source: BMC Bioinformatics. 2004 Oct 8;5:147. doi: 10.1186/1471-2105-5-147 (PMC528731; doi:10.1186/1471-2105-5-147)
Supplement: Additional File 2 — The original results of the above study (non-essential files are deleted to keep the file size under the limit set by BMC bioinformatics). [file 1471-2105-5-147-S2.bz2 › chilibotAdditionalFile2/dip50/407ID11468284E10068/html/CSF2_CSF3R.html]

 


 **CSF2** and **CSF3R** 
  
Found 185 abstracts in PubMed, retrieved 50.  
 

 What does Google say? 
 PDF only 
| .edu only 

---

**Interactive relationship** (e.g. stimulation, inhibition, etc)

**Stimulatory relationship**- Granulocyte colony stimulating factor  [ **CSF2** ]  G CSF is the major regulator of granulopoiesis and acts through binding to its specific receptor G CSF R  [ **CSF3R** ]  on neutrophilic granulocytes.  Ref: 11806990 Blood, 2002
- Granulocyte colony stimulating factor  [ **CSF2** ]  G CSF induces proliferation, maturation, and functional activities of myeloid progenitors and mature neutrophils through a specific receptor, the G CSF R  [ **CSF3R** ] .  Ref: 12586631 Blood, 2003
**Neutral relationship**- Here we show that expression of BCR ABL in myeloid precursor cells leads to transcriptional suppression of the granulocyte colony stimulating factor  [ **CSF2** ]  receptor G CSF R  [ **CSF3R** ]  encoded by  **CSF3R** , possibly through down modulation of C EBPalpha the principal regulator of granulocytic differentiation.  Ref: 11753385 Nat Genet, 2002

**Non-interactive relationship** (e.g. studied together, co-existance, homology, etc.)

- The granulocyte colony stimulating factor  [ **CSF2** ]  receptor G CSF R  [ **CSF3R** ]  forms a tetrameric complex with G CSF containing two ligand and two receptor molecules.  Ref: 11468284 J Biol Chem, 2001
- Overall, among 50 patients who completed the study, there were 3 erythroid responses as determined by complete abolition of red cell transfusions 1 4% in the G CSF r  [ **CSF3R** ]  EPO and 2 10.5% in the GM CSF  [ **CSF2** ]  r EPO treated groups.  Ref: 11146570 Haematologica, 2001
- To elucidate the function of Cb2 in myeloid leukemia cells, a set of in vitro experiments was carried out using 32D G CSF R  [ **CSF3R** ]  granulocyte colony stimulating factor  [ **CSF2** ]  receptor cells transfected with exon 1B exon 2 Cb2 complementary DNA and a myeloid cell line carrying a virus insertion in Cb2 ie, NFS 78.  Ref: 11929767 Blood, 2002
